# Optimization and visualization of the edge weights in optimal assignment methods for virtual screening

**DOI:** 10.1186/1756-0381-6-7

**Published:** 2013-03-26

**Authors:** Lars Rosenbaum, Andreas Jahn, Alexander Dörr, Andreas Zell

**Affiliations:** 1 University of Tübingen, Center for Bioinformatics (ZBIT), Sand 1, 72076 Tübingen, Germany

## Abstract

**Background:**

Ligand‐based virtual screening plays a fundamental part in the early drug discovery stage. In a virtual screening, a chemical library is searched for molecules with similar properties to a query molecule by means of a similarity function. The optimal assignment of chemical graphs has proven to be a valuable similarity function for many cheminformatic tasks, such as virtual screening. The optimal assignment assumes all atoms of a query molecule to be equally important, which is not realistic depending on the binding mode of a ligand. The importance of a query molecule’s atoms can be integrated in the optimal assignment by weighting the assignment edges. We optimized the edge weights with respect to the virtual screening performance by means of evolutionary algorithms. Furthermore, we propose a visualization approach for the interpretation of the edge weights.

**Results:**

We evaluated two different evolutionary algorithms, differential evolution and particle swarm optimization, for their suitability for optimizing the assignment edge weights. The results showed that both optimization methods are suited to optimize the edge weights. Furthermore, we compared our approach to the optimal assignment with equal edge weights and two literature similarity functions on a subset of the Directory of Useful Decoys using sophisticated virtual screening performance metrics. Our approach achieved a considerably better overall and early enrichment performance. The visualization of the edge weights enables the identification of substructures that are important for a good retrieval of ligands and for the binding to the protein target.

**Conclusions:**

The optimization of the edge weights in optimal assignment methods is a valuable approach for ligand‐based virtual screening experiments. The approach can be applied to any similarity function that employs the optimal assignment method, which includes a variety of similarity measures that have proven to be valuable in various cheminformatic tasks. The proposed visualization helps to get a better understanding of the binding mode of the analyzed query molecule.

## Background

High‐throughput screenings of a chemical library for ligands against a certain protein target play a fundamental part in the early stages of the drug discovery pipeline. The searching of a chemical library *in‐silico*, also called virtual screening (VS), is the complementary computational approach to high‐throughput screening [1]. The goal of a VS is to enrich molecules that are biologically active against a protein target in a preferably small top fraction of the ranked library. Only molecules with a top rank are then further analyzed with biological assays to validate their biological activity [2]. Using VS, a chemical library comprising several million molecules can be searched for the most promising compounds to test in an experimental screening.

The computational approaches can be divided into structure‐based and ligand‐based VS methods. Structure‐based approaches try to predict the conformation and orientation of potential ligands in the binding pocket of a target protein [3], which requires experimentally determined three‐dimensional coordinates of the protein structure. Ligand‐based VS methods rank a chemical library operating only on a small set of known ligands that serve as query structures. Hence, ligand‐based methods can still be applied if protein structures are not available [4].

In a ligand‐based VS experiment, the similarity between every library molecule and a query molecule is measured by some similarity function. Highly similar library compounds are assigned a top rank, whereas molecules with a small similarity to the query molecule are assigned a low rank. Thus, a ligand‐based VS experiment highly depends on the chosen similarity function.

A large variety of different similarity functions have been proposed in the last two decades and the development of new or improvement of existing functions is still a field of active research [5-7]. All similarity functions can be categorized into different classes regarding the representation of the molecules. A simple and fast approach encodes each molecule with a fingerprint like extended‐connectivity fingerprints (ECFP) [8] or MOLPRINT2D [9] and calculates the similarity between fingerprints with the well known Tanimoto coefficient. A more elaborate approach interprets the molecules as chemical graphs. The similarity between two chemical graphs can then be evaluated by means of maximum common subgraph approaches [10] or a variety of graph kernels [11]. Fröhlich et al. introduced the concept of an optimal assignment (OA) of arbitrary objects of two sets in the field of cheminformatics [12,13]. The OA similarity measure and its various extensions have proven to be a valuable approach in many cheminformatic problems, such as quantitative structure‐activity relationship predictions [14] and virtual screening [15,16].

The OA assumes every object, or atom in case of chemical graphs, to be of the same importance. Hence, all parts of a query molecule are equally important for the OA. However, depending on the binding mode of a ligand to its protein target, not all substructures of a ligand might be equally important for binding. A substructure of a ligand that exhibits important interactions (e.g. H‐bonds) with the protein target should contribute considerably more to the binding affinity of a ligand than a substructure that does not directly interact with the protein. Hence, the assumption of equally important atoms might not be realistic in a VS for ligands against a specific protein target.

This study has three aims. First, we present an extension to the existing standard OA method, which allows for assigning a different importance to each atom of a given query molecule. The importance is included by weighting the assignment edge that originates from an atom of the query molecule. The importance of the atoms of the query molecule can be determined by looking at the binding mode of the query if a crystal structure and expert knowledge are at hand. However, a crystal structure is often not available in the early stages of drug discovery or the docking of the query into an existing crystal structure is not satisfying. However, a data set of known ligands and decoys is usually available for ligand‐based VS.

The second aim of this study deals with optimizing the assignment edge weights for optimal VS performance on a data set of known ligands and decoys. Optimizing for optimal VS performance results in a non differentiable objective function with multiple local optima. Evolutionary algorithms for numerical optimization problems, such as a genetic algorithm for numerical problems (realGA), evolution strategies (ES) [17], particle swarm optimization (PSO) [18], and differential evolution (DE) [19] are capable of solving such optimization problems.

ES, realGA, and DE are evolutionary algorithms, which use mutation, crossover, and selection operators to search the minimum of a certain fitness function. The realGA employs mutation and crossover operators that are adapted to real‐valued problems in combination with a fitness value based selection. Evolution strategies employ mutation operators and a deterministic selection based on fitness ranks. DE is similar to realGA regarding selection and crossover, but uses a special mutation operator that is based on differences between population individuals. PSO belongs to the class of swarm intelligence algorithms, which use a set of particles that move with a certain velocity to search the minimum of a fitness function. In this study, we concentrated on DE and PSO because of their performance compared to realGA and ES on two real‐world optimization problems recently tackled by our group [20,21]. DE and PSO have also been successfully applied in other practical optimization tasks [22-24].

The third aspect of this study concerns the interpretability of the optimized edge weights because besides a good performance, the information, which parts of the molecule are important for a good retrieval of actives, is valuable for a medicinal chemist. The presented approach encodes each assignment edge weight by the radius of the atom it originates from in a Ball & Stick visualization.

The methodology of optimizing the assignment edge weights was presented at the EVOBio2012 conference [25]. Compared to the conference contribution this study addresses several aspects of the edge weight optimization in more detail, augments the method with a visualization, and compares the performance to two literature VS similarity functions.

The performance of our method and the other literature VS methods was evaluated on 13 VS benchmark data sets using sophisticated VS performance metrics for measuring both the overall performance as well as the early enrichment performance. Furthermore, we demonstrate the abilities of the visualization approach on one of the benchmark data sets.

The results show that the optimization of edge weights results in a considerable performance gain compared to the equally weighted OA and two other literature similarity functions. The visualization of the edge weights helps to understand which atoms of the query are important to obtain a good retrieval of active compounds.

## Methods

This section first gives a short introduction to the OA similarity measure and motivates the importance of the optimization of the edge weights. This part has already been presented in the conference contribution. Second, we present two evolutionary algorithms that are capable of optimizing the assignment edge weights. Finally, we discuss how the edge weights can be visualized to allow the interpretation of the optimization results.

### Optimal assignment similarity measure

**Figure 1:**
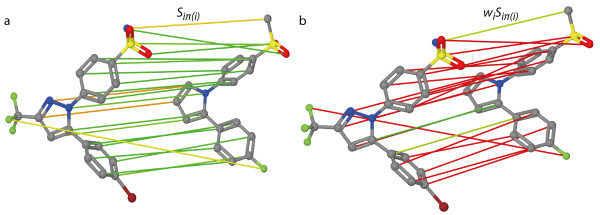
**Optimal assignment with equal and optimized edge weights.** Exemplary optimal assignment of two molecules of the COX2 dataset. The atom assignments are based on pairwise‐atom similarity calculations of the OA with equal edge weights (**a**) and with optimized edge weights (**b**). Green optimal assignment edges represent a high (weighted) atom similarity whereas red edges indicate a low (weighted) atom similarity.

The concept of the OA of two sets of arbitrary objects was introduced to the field of cheminformatics by Fröhlich et al. [12,13]. The OA similarity measure for molecular graphs calculates the similarity between the graphs by finding an optimal mapping of the atoms of the smaller molecule on a subset of the atoms of the larger molecule. A mapping of the atoms of two molecular graphs is called optimal if it maximizes the sum of pairwise atom similarities. An optimal mapping between two atoms of the graphs results in an optimal assignment edge as shown in Figure 1.

The first step of the OA approach is the calculation of the matrix *S* of pairwise inter‐molecule atom similarities. A pairwise atom similarity *S*_*ij *_is calculated by comparing the physio‐chemical properties of each atom with a radial basis function (RBF). The OA uses 24 atom and 8 bond descriptors calculated by the chemical expert system JOELib2 [26].

The local environment of an atom influences its physio‐chemical properties. Hence, the pairwise atom similarity calculation includes information of the topological neighbors and the bonds up to a predefined depth. The information is integrated by a recursive atom‐wise similarity calculation of the neighbors. The influence of the local environment is controlled by a decay parameter *d* because the influence of a neighboring atom decreases with its topological distance. In our experiments, we considered topological neighbors up to depth 2. The decay parameter *d*(*a*_*i*_,*nb*) of a topological neighbor *nb* of atom *a*_*i *_was calculated as given in Equation 1, where dist(*a*_*i*_,*a*_*nb*_) is the topological distance between the center atom *a*_*i *_and its neighboring atom *a*_*nb*_.

(1) $$d(a_{i},\text{nb})={\left(1-\frac{\text{dist}(a_{i},a_{\text{nb}})}{3}\right)}^{2}$$

Given two molecular graphs A and B with atoms $a_{1},\ldots ,a_{m}$ and $b_{1},\ldots ,b_{n}$ and the matrix *S* of pairwise atom similarities, the OA problem can be formulated as finding an optimal permutation *π* of indices that maximizes the objective function of Equation 2.

(2) $$S(A,B)=\left\{\begin{array}{cc} {\text{max}}_{\pi }\overset{m}{\underset{i=1}{\sum }}S_{\mathrm{i\pi}(i)} & \;\text{if}\;n>m\\ {\text{max}}_{\pi }\overset{n}{\underset{j=1}{\sum }}S_{\pi (j)j} & \;\text{otherwise} \end{array}\right)$$

Using the Hungarian method [27], an optimal solution for the OA problem can be computed in *O*(max(*m*,*n*)^3^).

The sum of all pairwise atom similarities in Equation 2 increases with the number of atoms that a molecule contains. To obtain comparable similarity values for molecules of arbitrary sizes the result *S*(*A*,*B*) of the OA is normalized to a range of [0,1] by Equation 3.

(3) $$S_{\text{OA}}(A,B)=\frac{S(A,B)}{\sqrt{S(A,A)S(B,B)}}$$

An example of an OA of two molecular graphs is visualized in Figure 1a.

### Optimal assignment edge weights

In a ligand‐based VS against a specific protein target, a single query is used to search a chemical library for other potentially active compounds. Generally, not all parts of the query are equally important for activity. The exact physio‐chemical properties of substructures that exhibit crucial interactions with the protein target should be more important and conserved than the properties of some linker region. Hence, important substructures should receive more attention in the OA procedure than unimportant substructures.

The different importance of the atoms of a query molecule can be represented in the OA approach by weighting the assignment edges that originate from the atoms. Atoms that are part of more important substructures result in larger edge weights whereas atoms within less important parts of the query molecule result in smaller edge weights. Consequently, the contribution of an assignment to the sum of pairwise atom similarities increases with the importance of the substructure that contains the assignment.

Assuming a fixed query molecule Q with atoms $q_{1},\ldots ,q_{m}$, the objective function of the OA problem (Equation 2) is modified to Equation 4.

(4) $$\begin{array}{ll} S(Q,B)= & \left\{\begin{array}{cc} {\text{max}}_{\pi }\overset{m}{\underset{i=1}{\sum }}w_{i}S_{\mathrm{i\pi}(i)} & \;\text{if}\;n>m\\ {\text{max}}_{\pi }\overset{n}{\underset{j=1}{\sum }}w_{\pi (j)}S_{\pi (j)j} & \;\text{otherwise} \end{array}\right)\; \end{array}$$

(5) $$\text{subject}\;\text{to}\;\overset{m}{\underset{i=1}{\sum }}w_{i}=m$$

The edge weights *w*_*i *_represent the importance of the corresponding atom of the query molecule. The edge weights are not optimized by the OA procedure. They are fixed throughout the similarity calculations of a complete VS run and need to be determined prior to the VS of a chemical library.

An OA with optimized edge weights is visualized in Figure 1b. Setting edge weights close to zero, can result in a very small weighted similarity (red in Figure 1b), even for highly similar atom environments (green in Figure 1a). Consequently, only a few assignments contribute to the sum of weighted similarities *S*(*Q*,*B*). The assignment in Figure 1b only has three assignment edges with a large weight. The focus on a few number of assignments can cause topological errors during the OA because only the correct mapping of the assignments with a large weight is important. However, an advantage is that the OA can focus on the mapping of important pharmacophores.

### Edge weight optimization

The assignment edge weights can be determined using expert knowledge of the exact binding mode of the query molecule to its target protein. However, depending on the availability of high quality crystal structures and literature, the exact binding mode might be unknown. Hence, the parts of a query molecule that are important for binding to the protein target are often unknown. Additionally, it is hard to assign exact numerical weights, even with expert knowledge. We propose to optimize the assignment edge weights for optimal VS performance on a data set of known ligands and decoys, which is usually available even in the early stages of drug discovery.

We employed two different types of evolutionary algorithms to optimize the edge weights of a given query structure. The VS performance on a data set of known ligands and decoys, which is called optimization data set throughout this paper, was used as fitness value for the optimization. The fitness value of a given set of weights was calculated by performing a VS run on the optimization data set. To ensure the weight constraints (5), the weights were bounded by [−0.5,0.5] during an optimization step and then normalized to suffice the constraints for the fitness evaluation.

Changing the edge weights, in particular driving weights close to zero, can dramatically change the OA. Changing the OA of several library compounds results in a different VS result. Thus, the fitness landscape contains multiple local optima. Evolutionary algorithms for numerical optimization with a good exploratory behavior should be used for optimization. A good exploratory behavior is crucial to avoid premature convergence to a local optimum, which could be observed for an ES with elitism in a practical study with a multimodal fitness function [20]. Besides a good exploratory behavior, an optimization algorithm should be able to keep a potential global minimum and do a local search around it, which is known as exploitation. A better exploration of the search space can improve the performance on a jagged fitness landscape. However, an algorithm with a good exploration of the search space, but a bad exploitation, can lose a previously found potential optimum. Hence, a balance between exploration and exploitation is desired.

DE and PSO are popular evolutionary algorithms for tackling numerical problems with multiple local optima or dynamically changing fitness functions [28,29]. Furthermore, realGA and ES without elitism are suited to optimize the assignment edge weights. Implementations of the aforementioned algorithms are available in the optimization framework EvA2 [30].

In a preliminary experiment we applied the optimizers realGA, ES, DE, and PSO with their EvA2 standard settings. The results indicated that realGA and ES performed worse than DE and PSO for the edge weight optimization. These results are in line with other practical studies performed by our group [20,21], which calculated fitness values using a complex or dynamically changing model. DE and PSO might perform better for such problems. However, this performance advantage and well performing parameter settings of the algorithms cannot be generalized as stated by the No Free Lunch Theorems for search [31].

#### Particle swarm optimization

PSO is a population‐based optimization technique inspired by swarms of fish or birds. Each individual, or swarm particle, *x*_*i *_is characterized by its position in the problem space and its current travel velocity *v*_*i*_(*t*) that allows it to move in the problem space. The particles are arranged in a logical topology which defines a neighborhood *N*(*x*_*i*_) for each particle *x*_*i*_. The motion of a particle is influenced by both individual knowledge and knowledge of neighboring swarm individuals.

At iteration *t*, a swarm particle *x*_*i *_is attracted by the best position $x_{i}^{h}$ in the particle’s history and the best position $x_{i}^{n}$ found by its neighboring particles’ history, resulting in Equations 6 and 7.

(6) $$\begin{array}{lll} v_{i}(t+1) & =\chi \left[v_{i}(t)+r_{1}\phi_{1}(x_{i}^{p}-x_{i}(t))\right)\; & \;\\ & \;+\left(r_{2}\phi_{2}(x_{i}^{n}-x_{i}(t))\right]\; \end{array}$$

(7) $$x_{i}(t+1)=x_{i}(t)+v_{i}(t+1)$$

The parameters *ϕ*_1 _and *ϕ*_2 _control the trade‐off between the different attractors. The update is performed by component wise sampling *r*_1_,*r*_2 _∼ *U*(0,1) for randomized exploration, where *U* is a uniform distribution. The constriction approach includes a constriction factor *χ* to assure that the swarm reliably converges [32]. Using the constriction approach, it is not necessary to limit the velocity vector to a maximum velocity *v*_*max*_. Commonly used neighborhoods are ring, 2D‐grid, or star topologies [33].

The employed neighborhood and the parameter *χ* have a major influence on the exploration and exploitation behavior of PSO. A smaller neighborhood results in a slower convergence, but it exhibits a better exploration of the search space. Larger values of *χ* produce larger velocities of the particles, which enhances the exploration of the search space. However, faster velocities result in a slower convergence. Additionally, a larger population increases the exploration of the search space.

#### Differential evolution

DE is an evolutionary algorithm that replaces the mutation operator of genetic algorithms with a differential operator. DE generates new candidates by recombining a population individual and a vector of differences between selected members of the population. There exist several DE variants, which differ in the calculation of the difference vector [19,28]. The nomenclature scheme to reference different DE variants is as follows: *DE*/*a*/*b*/*c*. In this nomenclature, *a* specifies the vector to be mutated, *b* defines the number of difference vectors used for mutation, and *c* defines the recombination scheme. In the following, the parameter *c* is omitted because we only used binomial recombination (*c*=bin).

Using binomial recombination, a new candidate solution $u_{i}=(u_{i,1},\ldots ,u_{i,m})$ is calculated from a population individual *x*_*i *_as given in Equation 8.

(8) $$\begin{array}{ll} u_{i,j} & =\left\{\begin{array}{c} x_{a,j}+F\cdot \overset{b}{\underset{k=1}{\sum }}(x_{r_{1}^{k},j}-x_{r_{2}^{k},j})\\ \;\;\text{if}\;U(0,1)\leq \text{CR}\;\text{or}\;j=j_{r}\\ x_{i,j}\;\;\;\text{otherwise} \end{array}\right)\; \end{array}$$

The vector to be mutated *x*_*a *_is a random population individual for *a*=“*rand*”, the current best individual for *a*=“*best*”, and *x*_*i*_+*λ*(*x*_*best*_−*x*_*i*_) for *a*=“*current‐to‐best*”. The parameters *F*,*λ*∈ [0.3,0.9] control the amplification of the differential variation and the parameter *CR *∈ [0,1] represents the crossover rate. The vectors $x_{r_{1}^{k}},x_{r_{2}^{k}},k=1,\ldots ,b$ are randomly chosen, mutually exclusive population individuals. The randomly chosen index *j*_*r *_ensures that at least one position is recombined. In contrast to the version of *DE/current‐to‐best/1* described by Mezura‐Montes et al. [28], the EvA2 version of the variant *DE/current‐to‐best/1* uses a combination of probabilistic and arithmetic recombination.

The chosen mutation variant and the parameters *CR* and *F* influence the exploration and exploitation behavior of DE. The variant *rand* uses a random population individual for mutation, which results in a better exploratory behavior, but the variant can take long to converge. The variant *best* uses the best population individual, which can limit the exploration of the search space and result in premature convergence. Higher *CR* and *F* values result in a higher mutability, which enhances the exploration. At the same time, a higher mutability might limit the search around a potential global minimum. As for PSO, a larger population results in a better exploration.

### Visualization of edge weights

The assignment edge weights represent the importance of the atoms of the query molecule. Due to the integration of local atom environments in the OA procedure, a large edge weight means that in addition to the corresponding atom itself the local environment around the atom is important. Hence, an edge weight needs to be redistributed between the corresponding atom and its local environment according to the decay parameter (1) of the atom similarity calculations.

The redistribution of an edge weight *w*_*i *_of an atom *a*_*i *_needs two passes over the atoms local environment. In the first pass, the sum of all decay parameters *d*_*tot *_is calculated as given in Equation 9, where *N*(*a*_*i*_) is the set of topological neighbors up to depth 2.

(9) $$d_{\text{tot}}=\underset{\text{nb}\in N(a_{i})}{\sum }d(a_{i},\text{nb})$$

In the second pass, the weight *w*_*i *_is distributed among the neighbors *nb* according to Equation 10, where **w**’ is the new weight vector. The new weight vector **w**’ is initialized with zero before the second pass. The update (10) is successively performed for all atoms *a*_*i*_.

(10) $$w_{\text{nb}}^{\prime }=w_{\text{nb}}^{\prime }+\frac{w_{i}\cdot d(a_{i},\text{nb})}{d_{\text{tot}}}\;\forall \;\text{nb}\in N(a_{i})$$

After the distribution of the weights, the new weight vector **w**’ is normalized to [0,1] to avoid visualization problems for large weights. Then, the weight $w_{i}^{\prime }$ of an atom can be visualized by the ball size in Å in a Ball & Stick molecule visualization. A large ball size indicates a larger weight whereas a small ball size indicates a smaller weight.

## Experimental

### Data sets

We evaluated our approach on a wide range of pharmaceutically relevant targets using a subset of the Directory of Useful Decoys (DUD) Release 2 [34,35]. The DUD contains known actives and mimetic decoys for 40 protein targets. The DUD was designed to serve as an unbiased, publicly available benchmark database for the evaluation of docking methods. Due to a possible analogue enrichment bias, the original DUD is not suited for ligand‐based VS experiments [36].

Thus, we applied the same preprocessing step as in the study of Jahn et al. [15] to make the data sets more suitable for ligand‐based VS. In the preprocessing step, a lead‐like filter as suggested by Good and Operea [37] was applied to the data sets and the active structures were clustered [38]. Each cluster represents a different chemotype or scaffold, which allows for the analysis of the enrichment of structurally diverse compounds.

We used the same 13 filtered subsets of the DUD as Jahn et al. because these data sets contain a sufficient number of different chemotypes. On a data set with a low number of scaffolds the VS performance would be mainly based upon a trivial enrichment. An overview of the data sets, their corresponding protein targets, and the number of clusters can be found in Table 1.

**Table 1:** Filtered DUD data sets used for the VS experiments

**Target**	**Target protein**	**Actives**	**Decoys**	**Clusters**
ACE	Angiotensine‐converting enzyme	46	1796	19
AChE	Acetylcholinesterase	100	3859	18
CDK2	Cyclin‐dependent kinase	47	2070	32
COX2	Cyclooxygenase‐2	212	12606	44
EGFr	Epidermal growth factor receptor	365	15560	40
FXa	Factor Xa	64	2092	19
HIVRT	HIV reverse transcriptase	34	1494	17
InhA	Enoyl ACP reductase	57	2707	23
P38	P38 mitogen activated protein	137	6779	20
PDE5	Phosphodiesterase 5	26	1698	22
PDGFrb	Platelet derived growth factor receptor kinase	124	5603	22
SRC	Tyrosine kinase	98	5679	21
VEGFr2	Vascular endothelial growth factor receptor	48	2712	31

Overview of the 13 filtered DUD data sets containing the target and the number of actives, decoys, and different chemotype clusters.

In contrast to docking methods, ligand‐based VS methods require a biologically active query molecule. In line with other VS studies on the DUD data sets [15,39], we used the ligands of the complexed crystal structures that were used to identify the binding sites for docking algorithms as query molecules.

### Evaluation of VS performance

The evaluation of a VS experiment is an important but challenging task, which has been discussed thoroughly in literature [40-42]. A plethora of sophisticated evaluation metrics have been suggested for measuring the performance of VS experiments, each with its strengths and weaknesses [43]. According to recommendations for assessing the VS performance [41], the VS results should be analyzed regarding three different aspects.

First, the performance on the complete data set should be considered. The overall performance can be measured by the well known area under the ROC curve (AUC). The receiver operator characteristics (ROC) curve plots the fraction of molecules correctly predicted as active (true positive rate or sensitivity) against the fraction of inactive molecules that are falsely predicted as active (false positive rate or 1‐specificity) for every possible decision threshold. The AUC can achieve values in the interval [0,1].

The second aspect is the early enrichment of actives, which originates from the situation in real‐world applications. Usually only a small fraction of hits of a VS experiment is further evaluated in experimental screens due to time and cost requirements. Consequently, it is important to enrich actives within the first few percent of a data set. We employed two different popular metrics, the BEDROC score and the ROC enrichment, to measure the early enrichment performance. The BEDROC score [44] augments the AUC with a decreasing exponential weighting function that reduces the influence of lower ranked structures. Hence, the amount a structure contributes to the final BEDROC score decreases exponentially with its rank. The weighting function is controlled by a parameter *α*. High values of *α* increase the importance of top ranked structures. In our experiments, we set *α*=53.6, which means that 80% of the final BEDROC score is based on the AUC performance in the first 3% of the ranked data set. The BEDROC score adopts values in the range [0,1].

The second employed early enrichment metric, the ROC enrichment (ROCE), measures the performance at a predefined false positive rate or top fraction of true decoys. The ROCE is calculated as given in Equation 11, where *N*_actives _is the number of actives and $N_{\text{actives}}^{\mathrm{x\%}}$ is the number of actives found along with the top *x%* of the true decoys. *N*_decoys _and $N_{\text{decoys}}^{\mathrm{x\%}}$ are defined analogously for the decoys. The ROCE is essentially the sensitivity SE_*x%*_ divided by the predefined false positive rate FPR_*x%*_ of *x%*.

(11) $${\text{ROCE}}_{\mathrm{x\%}}=\frac{{\text{SE}}_{\mathrm{x\%}}}{{\text{FPR}}_{\mathrm{x\%}}}=\frac{\frac{N_{\text{actives}}^{\mathrm{x\%}}}{N_{\text{actives}}}}{\frac{N_{\text{decoys}}^{\mathrm{x\%}}}{N_{\text{decoys}}}}$$

The final aspect for characterizing the performance of VS methods is the evaluation of the retrieval of different scaffolds. The enrichment of structurally diverse compounds, also called chemotype enrichment, is important for a pharmaceutical company. Some scaffolds might exhibit unwanted properties and only compounds with a substantially different scaffold compared to existing treatments can be patented [45]. All chemotype enrichment metrics are based on the clustering of actives into different chemotypes, which is then integrated in the ROC calculation [46,47]. As proposed by Mackey et al. [47], we employed the arithmetic weighting scheme. This scheme assigns a weight to each active compound which is inversely proportional to the number of compounds in its cluster. The weighting scheme is integrated into the ROCE by an arithmetically weighted sensitivity ${\text{SE}}_{\mathrm{x\%}}^{\text{aw}}$ as given in Equation 12. *N*_clusters _is the number of clusters, *N*_*j *_is the number of actives in the *j*‐th cluster, $\alpha_{\text{ij}}^{\mathrm{x\%}}$ is an indicator variable, which is 1 if the *i*‐th active of the *j*‐th cluster was found along with the top *x%* of true decyos, and $w_{\text{ij}}=\frac{1}{N_{j}}$ is the weight of an active structure. We refer to the arithmetically weighted ROCE as awROCE.

(12) $${\text{SE}}_{\mathrm{x\%}}^{\text{aw}}=\frac{\overset{N_{\text{clusters}}}{\underset{j}{\sum }}\overset{N_{j}}{\underset{i}{\sum }}\alpha_{\text{ij}}^{\mathrm{x\%}}w_{\text{ij}}}{N_{\text{clusters}}}$$

### Multirun stability and convergence speed

Due to the heuristic nature of evolutionary algorithms, the performance of several multirun optimizations can differ substantially. The stability throughout several multiruns is a desired property because the more stable an algorithm is the fewer multiruns are needed to achieve a good optimum, which results in lower runtime requirements. Generally, the performance on different splits of a data set varies more than the performance of several multiruns on a certain split. To obtain a sensible measure for the multirun stability we calculated the standard deviations for each split separately. The resulting deviations were then averaged over the different splits of a data set.

The performance on the different splits and data sets can vary substantially. To take the different performance into account, we also calculated the relative standard deviation (%RSD), which normalizes the standard deviation of a certain split of a data set by the mean performance on this split. However, the %RSD showed a stronger correlation with the mean performance (−0.60) compared to the standard deviations (−0.42). Hence, we decided to use the standard deviations as a measure for multirun stability.

Another important factor when optimizing a certain problem is the convergence speed of the evolutionary algorithm. The fewer fitness evaluations are needed, the faster the runtime. To evaluate the convergence speed, we ran each evolutionary algorithm until a large, fixed number of fitness evaluations was reached. The convergence point of a optimization run was defined as the number of fitness evaluations that are necessary to obtain a performance that is within a range of 1% of the best performance found after termination.

### Literature similarity functions

We employed two different literature similarity functions to compare the performance of our approach. First, the ECFP [8] with a Tanimoto coefficient, which calculates the similarity using 2D fingerprinting information. Second, the 4D flexible atom‐pairs similarity measure (4DFAP) [16,48], which is a sophisticated method that efficiently compares conformational ensembles of molecules.

The ECFP encodes the molecular graph of a compound as a binary fingerprint vector, where each bit of a fingerprint indicates the presence or absence of a molecular fingerprint feature. Each feature of an ECFP represents a circular substructure around a center atom. The algorithm starts with a set of initial atom identifiers for each atom and iteratively updates those identifiers similar to the Morgan algorithm [49]. In each iteration an atom identifier integrates the information of the neighboring atom identifiers and updates itself. Thus, with an increasing number of iterations an atom identifier contains informations from further and further away. The atom identifiers during each iteration are stored as an ECFP feature. The algorithm proceeds until a certain iteration limit is reached or until no new identifiers can be found. The similarity between two binary ECFP fingerprints can be calculated with the well known Tanimoto coefficient as presented in Equation 13.

(13) $$\text{Tan}(A,B)=\frac{\left|A\cap B\right|}{\left|A\cup B\right|}$$

The 4DFAP, a 4D‐based similarity measure, efficiently compares the conformational space of two given molecules. The approach can be divided into a preprocessing step and the actual similarity calculation.

In the preprocessing step, a conformational ensemble of a molecule is efficiently encoded by means of generative models. First, a conformational ensemble of a molecule *M* is generated. From the conformational ensemble the distance profiles of the $\frac{\left|M|(|M|-1\right)}{2}$ atom‐pairs are extracted. For each flexible atom‐pair, the distance profile is stored as a Gaussian‐mixture model. The parameters of the Gaussian‐mixture models are trained by a modified Expectation Maximization algorithm.

After the preprocessing, the actual similarity calculation between two molecules can be conducted. It operates on both the molecular graph and the precomputed generative models of the flexible atom‐pairs. Similar to the OA similarity measure, it first computes a matrix *S* of pairwise inter‐molecule atom similarities *S*_*ij*_. A pairwise atom similarity *S*_*ij *_is calculated by comparing the intra‐molecule atom‐pairs of two molecules. The rigid atom‐pairs are compared by a Tanimoto similarity function and the flexible atom‐pairs are compared by calculating the similarity between the stored GMM models. The final similarity is calculated by performing an OA on the matrix *S* of pairwise atom similarities. In principle, our approach of optimizing the edge weights could also be applied to the final step of the 4DFAP similarity measure.

### Evaluation setup

To robustly evaluate our method, we generated 10 randomized 50/50 splits. The first half of a data set was used as optimization data set to optimize the assignment edge weights for optimal VS performance. In each fitness evaluation, a complete VS run was performed and evaluated on the first half of a data set. The second half of a data set was used as external test set to obtain 10 VS results for unbiased performance evaluation. The literature similarity measures were also evaluated on these external test sets.

We performed 10 multiruns for each optimization. Each optimization was terminated after 15000 fitness evaluations, which were enough evaluations for each tested algorithm to converge. Due to the runtime requirements of 1.5 million fitness evaluations per data set, we evaluated the evolutionary algorithms only with their EvA2 default parameters.

We employed the constriction PSO implementation of EvA2 with its default parameters: *ϕ*_1 _= *ϕ*_2 _= 2.05, *χ *≈ 0.73, an initial velocity *v *= 0.2, and a population size of 30 individuals. These parameter settings worked well in earlier problems solved with PSO [20,50]. As neighborhood, we used a 2D‐grid with range two.

We evaluated the three DE variants *DE/rand/1*, *DE/best/2*, and *DE/current‐to‐best/1* with the Eva2 default parameters: *F *= 0.8, *CR *= 0.6, and *λ *= 0.6. The population size was set to 30 individuals. The population size is considerable smaller than suggested in a DE parameter study [51], which might limit the exploration of the search space. However, this size has proven useful in a recent study with a similar number of dimensions [20].

## Results and discussion

The results section is divided in four different subsections. The first subsection compares four different evolutionary algorithms for their suitability for optimizing the assignment edge weights. Then, the influence of the optimized VS performance measure is discussed. The third part compares the OA with optimized edge weights to the equally weighted OA and two literature VS similarity functions. The final subsection demonstrates the interpretability of our approach by visualizing the edge weights of the query of the InhA data set.

### Comparison of optimizers

We evaluated the four different evolutionary algorithms PSO, *DE/rand/1*, *DE/best/2*, and *DE/current‐to‐best/1* regarding VS performance, multirun stability, and convergence speed.

We assessed both the overall performance and the early enrichment performance of the four algorithms on the optimization split. The AUC performance was used to evaluate the overall performance. As an early enrichment metric we applied the BEDROC score. The AUC and BEDROC performance were obtained from the best multirun after optimizing for a maximum AUC and BEDROC score, respectively. While the performance on an external test set should be used to compare different models, the optimization performance is suited to compare the capability of the evolutionary algorithms to find the optimum of a given problem. Hence, we compared the optimization performance of the different algorithms.

**Figure 2:**
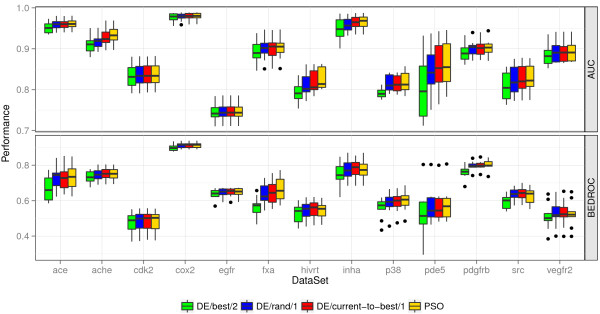
**Performance of optimization methods.** AUC and BEDROC performance of the different optimization methods on the 13 filtered DUD data sets. The performance was evaluated on the 10 optimization splits of a data set.

Figure 2 presents the performance of the four different optimizers. PSO, *DE/rand/1*, and *DE/current‐to‐best/1* yielded both a comparable AUC and BEDROC performance, whereas *DE/best/2* performed worse than the other three optimizers. Depending on the data set there also exist performance differences between the three comparable optimizers. With an average rank of 1.27, the PSO yielded the best AUC performance, followed by *DE/current‐to‐best/1* and *DE/rand/1* with an average rank of 1.88 and 2.85, respectively. The DE variant *DE/best/2* showed the worst average AUC performance with an average rank of 4.00. Similar relative rankings of the algorithms could be observed for the BEDROC performance.

While similar results concerning the performance of PSO and DE were obtained in a study that optimizes the parameters of metabolic networks [21], comparative studies between PSO and DE on various benchmark problems suggest that DE often performs better than PSO [52,53]. However, the global optimization performance of DE depends a lot on the parameter settings [51]. Increasing the population size might improve the performance of the DE variants.

Both *DE/rand/1* and *DE/current‐to‐best/1* performed better than *DE/best/2*. The weights of the multiruns of an iteration exhibit considerable differences even for multiruns with a similar performance, which indicates that the optimization problem is multimodal with several local optima. The variant *DE/best/2* has problems to efficiently explore the search space because of the recombination with the best individual of the population. Mezura‐Montes et al. [28] showed that *DE/current‐to‐best/1* exhibits this problem for multimodal problems due to the arithmetic recombination. Including a probabilistic recombination in the variant improves the exploratory behavior.

To evaluate the multirun stability of the evolutionary algorithms, we calculated the average multirun standard deviation of an algorithm when optimizing the AUC and the BEDROC performance. The optimized AUC and BEDROC performance on the optimization split was used for the stability calculations when optimizing the AUC and BEDROC, respectively.

**Figure 3:**
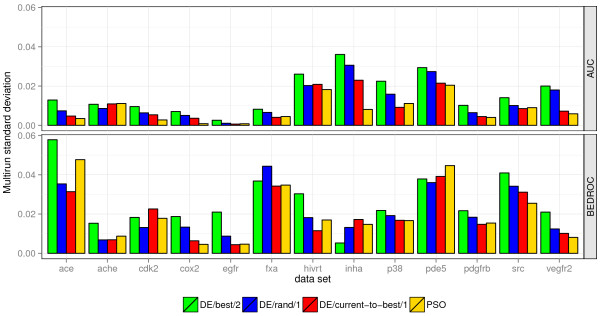
**Multirun stability of optimization methods.** Multirun stability of different optimization methods on the 13 filtered DUD data sets when optimizing the AUC or BEDROC performance. The optimized performance measure was used for the calculation of the standard deviations.

The multirun stability of the optimizers are presented in Figure 3. The results indicate that the AUC performance is more stable than the BEDROC performance over several multiruns. 80% of the BEDROC performance is calculated based on the performance in the first 3% of the ranked data set. Thus, for small data sets, the BEDROC score is based on the ranking of a few dozen compounds. A small change in the ranking, which can occur when slightly changing the weights, can have a considerable influence on the BEDROC performance. This behavior has an impact on the fitness landscape, which should be more jagged when optimizing the BEDROC. Consequently, the BEDROC performance fluctuates more throughout several multiruns.

With an average rank of 1.54 and 1.85, PSO and *DE/current‐to‐best/1*, respectively, were most stable throughout the AUC optimizations. *DE/rand/1* and *DE/best/2* behaved less stable on several data sets, which resulted in an average rank of 2.77 and 3.85, respectively. When optimizing the BEDROC performance, the PSO, *DE/current‐to‐best/1*, and *DE/rand/1* resulted in a similar multirun stability with an average rank of 2.08, 2.00, and 2.46, respectively. With an average rank of 3.46, *DE/best/2* also exhibited the worst stability when optimizing the BEDROC. The problem to efficiently explore the search space is a reason for the lower multirun stability of *DE/best/2*. The DE variant tends to converge to different local optima more easily than the other tested evolutionary algorithms.

**Figure 4:**
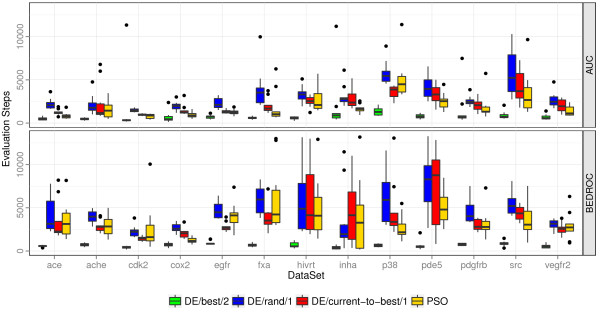
**Convergence speed of optimization methods.** Convergence speed of different optimization methods on the 13 filtered DUD data sets. An algorithm converged if the fitness is within a range of 1% of the best fitness after 15000 fitness evaluations.

The convergence speed of the algorithms was analyzed for optimizing the AUC as well as the BEDROC score. The convergence point is defined as the number of required fitness evaluations to obtain a performance that is within a range of 1% of the best result after 15000 fitness evaluations. For each data set, we averaged the convergence speed of the best multirun of each iteration. The convergence speeds are visualized as boxplots in Figure 4.

The convergence speeds indicate that 15000 fitness evaluations were enough evaluations for the algorithms to converge. Concerning the optimizers, *DE/best/2* exhibited the fasted convergence with an average rank of 1.00 regardless of the optimized performance measure, whereas *DE/rand/1* converged most slowly with an average rank of 4.00 and 3.77 when optimizing the AUC and BEDROC, respectively. PSO and *DE/current‐to‐best/1* achieved an average rank of 2.15 and 2.85, respectively, when optimizing the AUC performance. When optimizing the BEDROC score, PSO and *DE/current‐to‐best/1* exhibit a similar convergence behavior with an average rank of 2.54 and 2.69, respectively. The convergence speeds of the different DE variants are in line with a study on several benchmark functions by Mezura‐Montes et al. [28]. Their study showed that on average DE/best/* exhibits the fastest convergence. In our study, *DE/best/2* converged fastest because it converged to suboptimal local optima, which resulted in a lower performance.

On average, all algorithms, but *DE/best/2*, take between 45% and 80% more evaluation steps to converge when optimizing the BEDROC score, whereas *DE/best/2* needs 29% less evaluation steps. A possible explanation for this could be that the convergence point of 1% within the range of the best solution after 15000 evaluation steps is more stringent for the BEDROC score than for the AUC because the BEDROC performance is generally lower than the AUC performance. However, similar results were obtained with a fixed range of 0.01. The differences between the convergence speeds underline the assumption that the problem of optimizing the BEDROC score exhibits a more jagged fitness landscape. The variant *DE/best/2* converges faster because it hits a local optimum faster in a fitness landscape with more local optima.

The analysis of performance, multirun stability, and convergence speed favored PSO and *DE/current‐to‐best/1* for optimizing the assignment edge weights. Both algorithms exhibit the best performance and multirun stability combined with a reasonably fast convergence speed. Good multirun stability and fast convergence speed allow for the reduction of the number of fitness evaluations and multiruns in a productive environment to speed up the runtime while maintaining the performance. The following analyses are limited to PSO because it is one of the two suitable optimizers.

### Influence of the optimized VS metric

The assignment weights can be optimized with respect to any VS metric. The fitness function and with it the resulting model considerably depends on the employed VS metric. Hence, optimizing a certain metric can influence the model performance with respect to another VS metric. To evaluate the influence of the optimized metric, we performed experiments optimizing the AUC, a measure for the overall performance, and the BEDROC score, an early enrichment metric.

**Figure 5:**
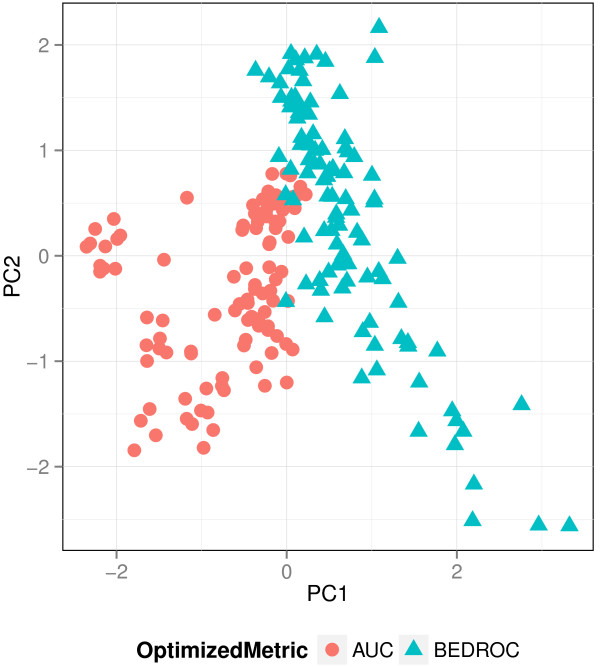
**PCA applied to FXa query weights.** Visualization of the weights of all iterations and multiruns of the FXa data set when optimizing for AUC and BEDROC performance.

**Figure 6:**
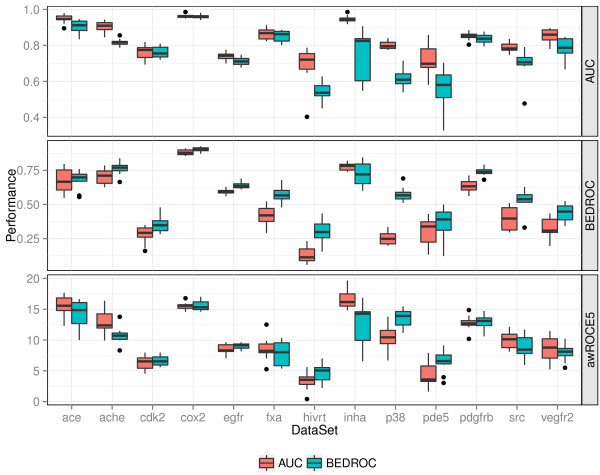
**Performance of AUC and BEDROC optimized queries+.** AUC, BEDROC and awROCE_5%_ performance of the AUC and BEDROC optimized queries on the external splits of the 13 DUD data sets.

To analyze if optimizing for the two metrics results in different weighting schemes, we visualized the weights with a principal component analysis (PCA), a dimension reduction technique. The PCA transforms the original weight matrix of all iterations and multiruns in a new orthogonal space, where each projection, also called principal component (PC), is a linear combination of the original weights. Figure 5 presents the results of a PCA applied to the FXa data set, a data set on which the performance of the queries differed with the optimized metric (see Figure 6). The weights that were optimized with the same VS metric clearly cluster. This behavior can even be observed when the performance difference between the VS metrics is marginal.

To analyze the effect of the two optimization metrics on the performance, we compared the performance of the optimized queries with respect to the AUC, BEDROC, and awROCE_5%_ performance on the external test splits. The results are presented as boxplots in Figure 6. Optimizing the AUC improved the overall performance of the optimized query. The AUC optimized queries resulted in a better AUC performance on all of the benchmark data sets. Vice versa, optimizing the BEDROC score resulted in a better BEDROC performance compared to optimizing the AUC. The BEDROC optimized queries achieved a better median BEDROC performance on all but one data set. However, both the BEDROC and AUC optimized queries result in a similar awROCE_5% _performance on most of the data sets.

The reason for the difference between the two early enrichment metrics BEDROC and awROCE_5% _is twofold. First, the BEDROC optimized queries tend to enrich molecules of larger chemotype clusters, which is penalized in the awROCE metric. Molecules of large clusters are likely to occur in the optimization split and the queries tend to be optimized for molecules of these clusters when focusing on an early enrichment. Thus, optimizing for the BEDROC score can result in weights that are applicable for a smaller number of clusters compared to optimizing for the AUC performance. This fact is supported by a better ROCE_5% _performance of the BEDROC optimized queries. Unlike the awROCE, the ROCE metric does not penalize large clusters. On the FXa data set, the ROCE_5% _performance favored the BEDROC optimized query whereas the awROCE_5% _performance slightly favored the AUC optimized query. A detailed analysis of the result table of the split with the most differing awROCE_5% _performance reveals that both optimized queries enriched an equal number of actives along with the top 5% of true decoys. However, the BEDROC optimized query mainly enriched actives from one of the largest clusters whereas the AUC optimized query also enriched a diverse set of smaller clusters.

**Figure 7:**
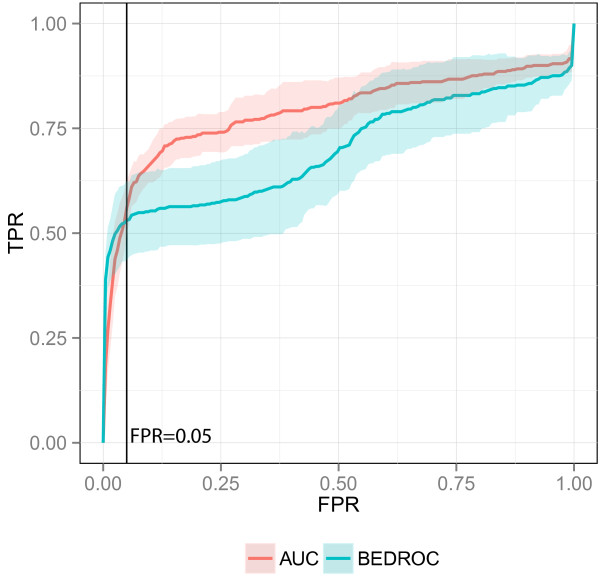
**ROC plot on SRC.** Average ROC of the AUC and BEDROC optimized query on the SRC data set. The vertical line indicates a true decoy fraction of 5%.

The second reason for a different BEDROC and awROCE_5% _performance is the percentage of the ranked data set that is taken into account by the metric. 80% of the BEDROC sore is based on the first 3% of the data set whereas the awROCE_5% _is based on the enrichment along with the top 5% of true decoys, which is more than 5% of the data set. Optimizing for an improved enrichment at 3% of the data set can yield an equal or worse early enrichment at 5% of the ranked data set as indicated by the average ROC on the SRC data set (Figure 7). On the SRC data set, the BEDROC optimized query achieved a better enrichment until a true decoy fraction of 5%. After this fraction, the AUC optimized query enriched more actives.

The analysis of the AUC and BEDROC optimized queries showed that the optimized VS metric has indeed a substantial influence on the resulting weights and the performance. Thus, care should be taken to select the correct optimization metric. Optimizing the AUC results in a good overall performance, a reasonable early enrichment, and a diversity of chemotypes for most of the data sets. Hence, it should be the default option for the optimization step. Employing the BEDROC score as optimization metric can lead to a substantially better early enrichment. However, this improved enrichment can come at the expense of a diversity of scaffolds or at the expense of the enrichment at a slightly larger fraction of the data set. Optimizing the awROCE would ensure a diversity of scaffolds, but it is not a very robust metric for optimization because of the strict cutoff at a certain fraction of true decoys.

We chose to use the AUC optimized query for the comparison between the OA with optimized edge weights and the literature similarity functions.

### Comparison to other similarity measures

In this subsection, we compare the OA with optimized edge weights (PSO‐OA) to its unoptimized pendant, the OA with equal edge weights (OA), and two literature similarity functions, an ECFP with a Tanimoto similarity coefficient (ECFP) and the 4D flexible atom‐pair kernel (4DFAP). The edge weights were optimized for the AUC performance using PSO. We evaluated the AUC, BEDROC, and awROCE_5%_ performance on the external test splits of the 13 filtered DUD data sets.

The results of the performance evaluations are compiled in Table 2. The statistical significance of the performance differences were tested with a Wilcoxon signed rank test. The PSO‐OA outperformed the OA on all data sets with respect to the AUC performance, which means that the optimization of edge weights considerable improved the performance on the complete data set. Compared to the literature similarity functions, the PSO‐OA yielded a better AUC performance on all data sets but the EGFr and the CDK2 data sets. On the CDK2 data set, the PSO‐OA achieved a comparable performance to 4DFAP, which performed best. These results yielded a best average rank of 1.23 for the PSO‐OA with respect to the AUC, followed by the 4DFAP similarity function with an average rank of 2.08. For the BEDROC scores, the PSO‐OA outperformed the literature similarity functions on 9 data sets and achieved a comparable performance on the PDE5 data set. Compared to the OA the PSO‐OA achieved a better performance on all but the HIVRT data set. The PSO‐OA exhibited a robust BEDROC performance with an average rank of 1.54, followed by the 4DFAP with an average rank of 2.31. Concerning the awROCE_5%_, the PSO‐OA outperformed the other approaches on 10 data sets and achieved a comparable performance on 2 data sets. Compared to the OA, the awROCE_5%_ performance was improved an all but the HIVRT and PDE5 data sets, on which the performance was comparable to the OA. The PSO‐OA and 4DFAP yielded the best results with an average rank of 1.31 and 2.54, respectively. The results demonstrate that the optimization of edge weights improves the performance on the complete data set as well as the early enrichment of different scaffolds compared to the original OA and two literature similarity functions.

**Table 2:** Performance of PSO‐OA and literature similarity functions

	**AUC**				**BEDROC**				**awROCE**_**5%**_			
**Target**	**PSO**	**OA**	**ECFP**	**FAP4D**	**PSO**	**OA**	**ECFP**	**FAP4D**	**PSO**	**OA**	**ECFP**	**4DFAP**
ACE	**0.947**	0.728	0.815	0.882	**0.678**	0.459	0.420	0.521	**15.6**	11.9	11.8	11.6
AChE	0.906	0.713	0.795	0.762	0.704	0.445	**0.788**	0.521	**13.0**	5.0	11.9	8.6
CDK2	*0.765*	0.521	0.510	0.776	**0.279**	0.162	0.116	0.212	**6.3**	3.0	1.7	4.0
COX2	**0.963**	0.879	0.853	0.892	**0.881**	0.630	0.627	0.810	**15.5**	8.4	7.3	11.7
EGFr	0.738	0.670	0.756	0.990	0.593	0.364	0.743	0.918	8.5	5.5	12.3	**17.8**
FXa	**0.865**	0.440	0.626	0.652	**0.420**	0.065	0.046	0.094	**8.5**	1.8	1.2	2.9
HIVRT	**0.690**	0.519	0.630	0.575	0.134	0.221	**0.316**	0.252	*3.3*	3.0	**3.9**	2.3
InhA	**0.946**	0.542	0.747	0.668	**0.777**	0.486	0.628	0.611	**16.7**	8.3	9.7	8.7
P38	**0.801**	0.444	0.266	0.674	**0.252**	0.093	0.096	0.070	**10.3**	4.0	1.7	3.0
PDE5	0.720	0.499	0.424	0.667	*0.308*	0.228	0.208	**0.351**	*4.5*	**5.5**	2.1	4.5
PDGFrb	**0.851**	0.481	0.437	0.640	**0.633**	0.216	0.139	0.145	**12.7**	4.4	3.7	4.1
SRC	**0.789**	0.454	0.356	0.496	**0.398**	0.138	0.001	0.066	**10.1**	4.7	0.0	3.1
VEGFr2	**0.853**	0.354	0.391	0.684	**0.329**	0.121	0.063	0.169	**8.6**	2.6	0.8	3.5
avg.rank	1.23	3.54	3.15	2.08	**1.54**	3.08	3.08	2.31	**1.30**	2.77	3.23	2.69

^1^AUC, BEDROC, and awROCE_5%_ performance of the PSO‐OA (PSO), OA, ECFP, and 4DFAP. **Bold** values indicate the best results on the corresponding data set and metric whereas *italics* indicate results that are statistically indistinguishable from the best result. The last row contains the average rank of the corresponding approach with respect to the metric and the other approaches.

**Figure 8:**
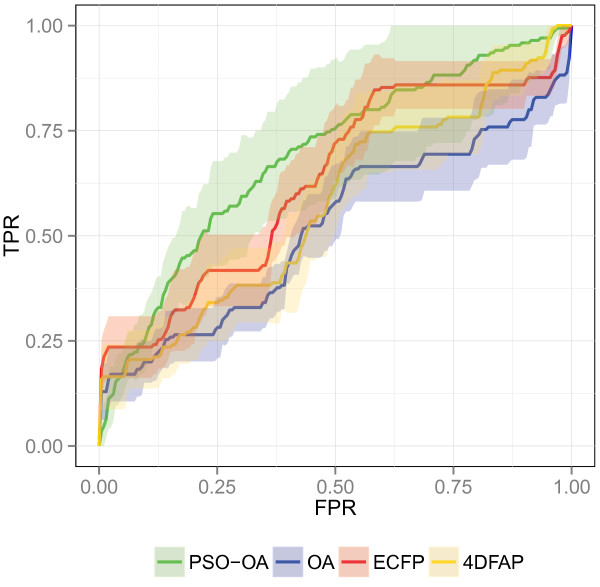
**ROC plot on HIVRT.** Average ROC of the different similarity functions on the HIVRT data set.

Overall, the optimization of edge weights yielded the worst results on the HIVRT data set. Compared to the OA the PSO‐OA was significantly outperformed only on the HIVRT data set with respect to the BEDROC performance. The ROC plot on the HIVRT data set (Figure 8) reveals that the OA enriched more actives up to true decoy fraction of 5%. At larger fractions of true decoys the PSO‐OA enriched more actives, which resulted in a better performance on the complete data set. Compared to the PSO‐OA, the ROC curves of the other approaches exhibit a more stepwise increase, which indicates that the approaches accumulate numerous molecules with the same chemotype before discovering a new chemotype. As a specific chemotype contains both actives and inactives, the scaffold‐wise accumulation leads to a more stepwise increase of the ROC curve. In contrast, the smoother curve progression of the PSO‐OA indicates that the method enriches molecules of different scaffolds. A similar behavior can be observed for other data sets. Thus, focusing on some optimal assignment edges allows for the enrichment of molecules with different scaffolds, also called “scaffold‐hopping”, which is important for a pharmaceutical company. Only substances with a substantially different scaffold compared to existing treatments can be patented [45].

**Figure 9:**
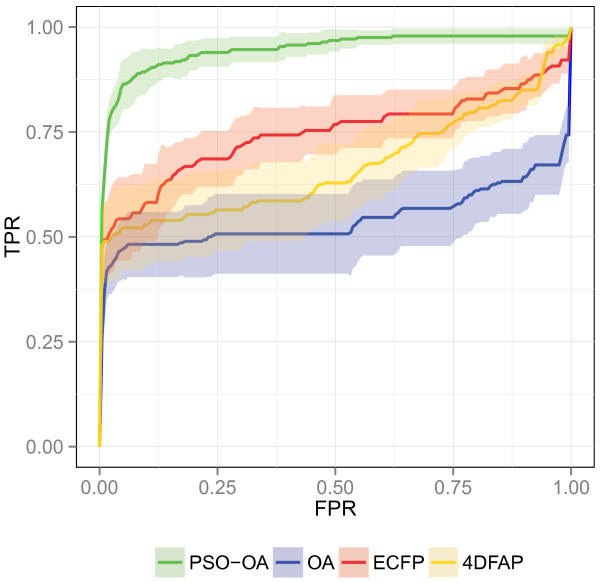
**ROC plot on InhA.** Average ROC of the different similarity functions on the InhA data set.

**Figure 10:**
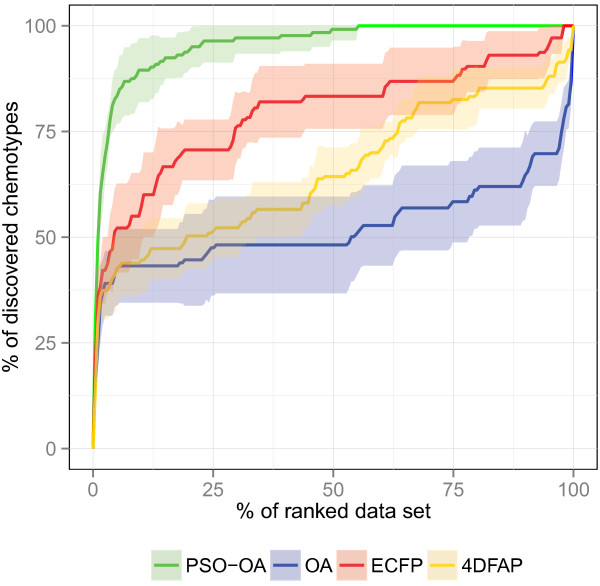
**Chemotype discovery on InhA.** Chemotype discovery of the different similarity functions on the InhA data set.

The ROC and chemotype discovery on the InhA data set are illustrated in Figures 9 and 10 respectively. A considerable performance gain of the PSO‐OA approach compared to the other approaches is apparent. The PSO‐OA retrieved more than 90% of the actives within 25% of the ranked data set (Figure 9). The other similarity functions, especially the OA, exhibit a much slower enrichment after retrieving ≈50*%* of the actives in the first few percent. 26 actives contain an indole attached to a carbonyl group, which is also present in the query structure. These actives are spread among 6 different clusters. The trivial retrieval of these compounds is mainly responsible for the enrichment of actives and the discovery of chemotypes in the first few percent of the data set. The optimization of edge weights encodes information in the query weights that enables the PSO‐OA to retrieve different scaffolds that do not contain the described substructure, which results in a considerable performance gain.

The results on the InhA data set indicate that the optimization of edge weights encodes a structure‐activity model of the molecules of the optimization data set in the query weights, which is not the case for the other similarity functions. Consequently, a considerable performance gain is to be expected. Perfectly fitting a structure‐activity model to the optimization data set might lead to substantial overfitting, a common problem of machine learning approaches. However, the fitness on the external test splits during the optimization runs as well as the final performance on the external splits indicate that overfitting was not a problem.

On the EGFr data set the 4DFAP outperformed all other approaches by a considerable margin. The 4DFAP was able to retrieve all actives within ≈30*%* of the data set whereas the PSO‐OA and the OA retrieved about 20% of the actives in the last percent of the data set. Hence, the conformational space encoding adds a significant amount of information for the EGFr data set [16]. While the PSO‐OA can improve the enrichment of actives compared to the original OA, it is restricted to the same 2D information as the OA, which limits the performance on the EGFr data set.

In principle, the optimization of edge weights can be applied to any optimal assignment approach. Thus, the weights in the optimal assignment step in the 4DFAP can be optimized with our approach. The atom similarity matrices of the 4DFAP are based on a different type of information (atom‐pair distances) compared to the OA (local atom similarities). Therefore, the optimized weights of the OA cannot be directly applied to the 4DFAP. Experiments with the optimized weights of our study (data not shown) resulted in a considerably reduced performance of the 4DFAP.

### Visualization of edge weights on InhA

In this subsection, we demonstrate and discuss the visualization of the optimized assignment edge weights on the InhA data set. The data set is among the three best performing data sets, which is crucial because a reasonable performance is necessary to obtain a sensible visualization. Furthermore, the weighted query of the InhA data set is suited to discuss the strengths and problems of the edge weight optimization and the visualization approach. For the visualization, we repeated the optimization of the edge weights with the complete data set. We performed 5 multiruns with 8000 fitness evaluations, which should be enough according to the multirun stability and the convergence speed of the PSO on the InhA data set. We employed the AUC as optimization metric.

The InhA data set contains ligands against the Enoyl ACP reductase, which is a promising protein target for the treatment of tuberculosis and malaria. The Enoyl ACP reductase mediates the reduction of fatty acid substrates using NAD^+^ as cofactor. As the DUD data sets were designed for docking, the binding mode of the query can be analyzed using the PDB entry 1P44, which contains the target protein complexed with the query.

**Figure 11:**
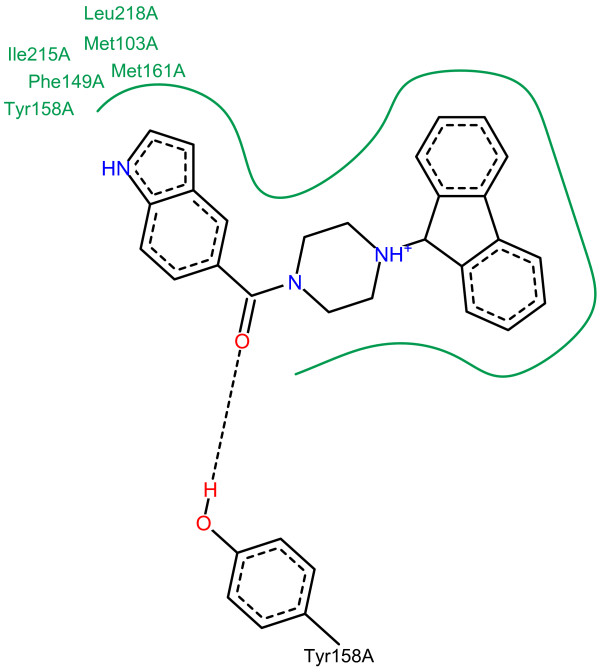
**Binding mode of the InhA query.** Binding mode of the query in the Enoyl ACP reductase visualized with PoseView [55].

Kuo et al. [54] describe several features that are important for the binding of the query to the protein target. As a competitor of the fatty acid substrate, the query mimics the substrates binding mode, which includes interactions with the cofactor NAD^+^. The fluorenyl group occupies a pocket that normally sees a long, hydrophobic acyl chain of the substrate. Consequently, a potential ligand needs a hydrophobic substructure that occupies this pocket assuming the same binding mode as the query. The fluorenyl group is connected to a carbonyl by a piperazine. Substitutions were not allowed for this piperazine due to steric clashes. The carbonyl group forms a hydrogen network with the cofactor NAD^+^ and a catalytic tyrosine (Tyr158) of the protein target. The described interactions, except for the hydrogen bonding with the cofactor NAD^+^, are visualized in Figure 11 using PoseView [55]. Finally, the indole nitrogen forms a weak hydrogen bond with the NAD^+^ cofactor.

**Figure 12:**
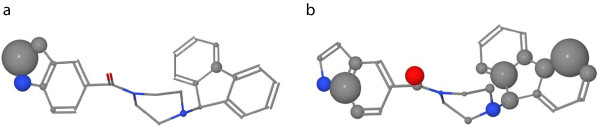
**Visualization of InhA query weights.** Visualization of the weighted query on the InhA data set. (**a**) shows the weights when using the complete data set for the optimization and (**b**) the weights when omiting a cluster of actives with a considerable different chemotype. An increasing atom radius represents an increasing importance of the atom.

**Figure 13:**
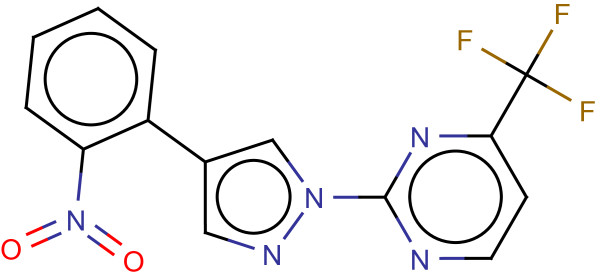
**InhA ligand without carbonyl.** InhA ligand ZINC03591544. The ligand is contained in a large cluster of actives that do not contain an indole connected to a carbonyl.

Figure 12a visualizes the optimized weights of the InhA query. Every important substructure, except for the carbonyl group, includes a group of atoms with increased weights. Most of the weight is placed on parts of a pyrrole because the indole that contains the pyrrole can be found in most of the actives. The weighting scheme of the pyrrole is mainly responsible for the superior performance compared to the other similarity functions. Most of the weighted similarity is based on a successful mapping of some of the pyrrole’s atoms. For a large cluster of actives that do not contain the indole connected to a carbonyl, the pyrrole’s atoms that have a large weight are mapped to parts of a pyrimidine ring. An example compound of this cluster can be found in Figure 13. This cluster and its analogues represent 15 of the 57 actives. These 15 ligands are also responsible for the fact that the carbonyl group does not receive any weight. Optimizing on a set, which omits the 15 ligands without a carbonyl group, results in a weighting scheme that assigns a considerable weight to the carbonyl as shown in Figure 12b. Additionally, the updated query of Figure 12b contains more atoms with an increased weight because the query does not have to enrich a large number of considerably different chemotypes.

The distribution of the weights critically depends on the ligands in the optimization set. If the 15 ligands without a carbonyl exhibit a completely different binding mode it would be less meaningful to force a fit between the query and those ligands. Such a forced mapping can result in a less sensible visualization, in our case the low weighted carbonyl group. An user of the visualization should keep in mind that increased edge weights represent atoms that need to be optimally assigned for a good retrieval of actives, which might not coincide with all features that are actually important for a specific binding mode.

**Figure 14:**
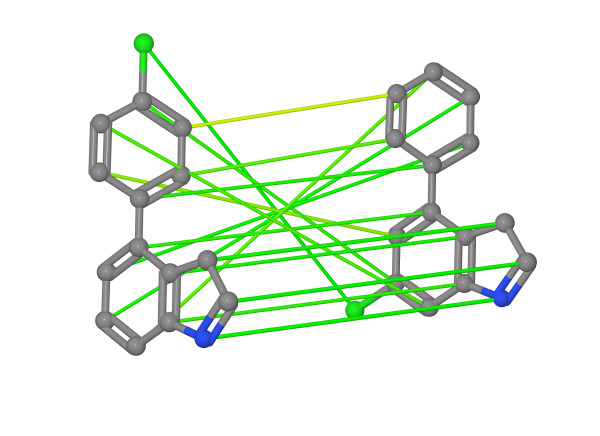
**Optimal assignment with topological errors.** Equally weighted optimal atom mapping on two phenylindole derivatives disclosing topological errors. 5 of the 6 intersecting edges map an atom of the indole on an atom of the phenyl or vice versa.

The ability to retrieve different scaffolds by focusing on a small number of assignment edges promotes a problematic behavior of the optimal assignment method. The optimal assignment maps each atom of the query onto an atom of the library compound. With an increasing number of atoms the risk of a topological error in the assignment step also increases. Topological errors are assignments that violate a substructure mapping, e.g. the atoms of a ring are assigned to the atoms different rings. Figure 14 shows a mapping that contains topological errors. This problematic behavior is promoted by increasing the weight of a few edges and by driving the remaining edge weights close to zero. As long as the atoms with an increased edge weight are mapped to appropriate atoms topological errors do not matter. This behavior could also be observed for the InhA query. Both an atom of the indole’s benzole part and an atom of the fluorenyl substructure were mapped to the nitrobenzene substructure of the compound depicted in Figure 13. While the mapping maximizes the final weighted similarity, it is questionable from a chemical point of view. Consequently one should always combine the visualization of the weights with an analysis of the quality of some of the assignments to retrieved library compounds.

There exist countermeasures to prevent the problem of an increased number of topological errors. First, pseudo weights can be added to the optimized weights, which would reduce the influence of large weights and increase the influence of weights that are close to zero in an optimization without pseudo weights. While pseudo weights might reduce the topological errors that were induced by a focus on a few large weights, they might also reduce the ability of discovering different chemotypes. The second countermeasure results from the general applicability of our method to any similarity function that employs the optimal assignment method. The 2‐step hierarchical assignment (2SHA) [15], a similarity function that was designed to prevent topological errors by doing a hierarchical assignment, can be subject to our optimization approach. A combination of both approaches might yield an improved VS performance with less topological errors.

## Conclusions

In this study we presented an extension of the optimal assignment method for chemical graphs that uses evolutionary algorithms to optimize the assignment edge weights with respect to a VS performance metric. In principle, the method is applicable to all similarity functions that employ the optimal assignment method like 4DFAP [16,48] or 2SHA [15]. Thus, the VS performance of a variety of VS similarity functions can be improved by optimizing the edge weights. An improved in‐silico VS performance, in particular an increased early enrichment, helps to increase the success rate of further biological assays and thus, reduces cost and time requirements. Additionally, we presented a visualization approach that allows for the interpretation of the optimized weights.

We evaluated 3 different DE variants and PSO for the suitability for the optimization of the OA edge weights. A suitable optimizer combines a good optimization performance, a reasonable multirun stability and a fast convergence speed. Both *DE/current‐to‐best/1* and PSO are suitable optimizers, but PSO achieved slightly better results than the DE variant.

Besides DE and PSO, other promising evolutionary algorithms can be applied for optimizing the assignment edge weights. An ES with covariance matrix adaptation (CMA‐ES) [56] or ant colony optimization (ACO) [57] could be used to solve the edge weight optimization problem. CMA‐ES and ACO are currently not available in the EvA2 framework, but the implementation and evaluation could be addressed in a further study. Additionally, GA or ES might perform better with different parameter settings.

The optimized VS metric has a considerable influence on the optimized weights. The results indicated that the optimization of the BEDROC score improves the early enrichment performance, whereas optimizing the AUC results in better overall performance and a better enrichment of different scaffolds. We advise to use the optimization of the AUC as default option.

We compared our method to the equally weighted OA and two other literature similarity functions on 13 DUD benchmark data sets. The results showed that the OA with optimized edge weights achieves a considerably better overall and early enrichment performance on a variety of pharmaceutically relevant targets. Particularly, our approach was able to perform so called “scaffold‐hops”, which is important for a pharmaceutical company.

The visualization approach was demonstrated on the weighted query of the InhA data set. The visualization showed that the optimization of edge weights yields sensible results with respect to the binding mode of the query. Thus, our approach helps medicinal chemists to get a better understanding of the binding mode of the query. However, if a large amount of compounds with a considerably different scaffold is present in the optimization set, the optimized weights might not completely coincide with the binding mode of the optimized query.

To conclude, we think that the optimization of edge weights combined with the visualization of the weights is a valuable tool for the VS of chemical libraries.

All employed programs are available free of charge as executable jar and source code at http://www.cogsys.cs.uni-tuebingen.de/software/OAOptimization/. This includes the library that was used for the optimization of the edge weights and a graphical user interface for the visualization of the results of an optimization.

## Competing interests

The authors declare that they have no competing interests.

## Authors’ contributions

LR designed and implemented the main part of the edge weight optimization, wrote the manuscript, participated in the design of the experiments and the discussion of the results. AJ participated in the implementation of the optimization, the design of the experiments, and the discussion of the results. AD participated in the design of the visualization and the discussion of the results. AZ supervised the study, participated in the design of the experiments, and discussed the results. All authors read and approved the final manuscript.
